# Impact of VP2 structure on antigenicity: comparison of BTV1 and the highly virulent BTV8 serotype

**DOI:** 10.1128/jvi.00953-24

**Published:** 2024-09-25

**Authors:** Sara L. Bissett, Polly Roy

**Affiliations:** 1 Department of Infection Biology, London School of Hygiene and Tropical, London, United Kingdom; University of Michigan Medical School, Ann Arbor, Michigan, USA

**Keywords:** BTV, VP2, tip domain, antigenicity, serotype, neutralizing antibody

## Abstract

Bluetongue virus (BTV) is an agriculturally and economically significant insect-borne virus that causes serious illness and death in sheep and other domestic and wild ruminants in large areas of the world. Numerous BTV serotypes exist, and distant serotypes exhibit unique neutralizing antibody profiles, which target the outermost capsid protein VP2. The predominant serotype-specific nature of the antibody response to VP2 is a barrier to the development of broad-spectrum prophylactic BTV vaccine candidates. Although VP2 is the main serotype determinant of BTV, the structural basis of serotype specificity has not been investigated. In this study, we utilized the recently available atomic structure of VP2 with a modeled tip domain to carry out *in silico* structural comparisons between distant serotypes BTV1 and BTV8. These analyses identified structural differences in the tip domain, positioned at the apex of VP2, and informed the design of mutant VP2 constructs. Dissection of tip domain antigenicity demonstrated that the region of structural difference between BTV1 and highly virulent BTV8 was a target of BTV neutralizing antibodies and that mutation of this region resulted in a loss of neutralizing antibody recognition. This study has for the first time provided insights into the structural differences, which underpin the serotype-specific neutralizing antibody response to BTV.

**IMPORTANCE:**

The immune system can protect against virus infection by producing antibodies, which bind and inhibit the virus from infecting the susceptible host. These antibodies are termed neutralizing antibodies and generally target the viral receptor binding protein, such as the VP2 of bluetongue virus (BTV). This pressure from the immune system can drive mutation of the viral protein resulting in escape from antibody-mediated neutralization and the evolution of serotypes, as is the case for BTV. Understanding the structural differences, which underpin the different BTV serotypes, could help guide the design of a BTV vaccine that targets multiple serotypes. In this study, we have mapped the VP2 structural differences between distant serotypes, to a region targeted by neutralizing antibodies, and have demonstrated for the first time how VP2 structure is the fundamental basis of serotype specificity.

## INTRODUCTION

Bluetongue virus (BTV) is a double-stranded RNA (dsRNA) virus belonging to the family *Sedoreoviridae*. BTV particles comprise two concentric layers of proteins, the inner core made up of VP3 and VP7, which harbors the 10 dsRNA segments of the viral genome, and the outer capsid, which contains the VP2 and VP5 proteins (1). VP2 is the most variable of the BTV proteins and the main determinant of virus serotype, harboring the majority of epitopes targeted by neutralizing antibodies (2, 3). VP2 also displays hemagglutination activity and initiates cell attachment by binding to α2,3- and α2,6-linked sialic acids, in a differential manner, which is dependent upon the cell type (4).

Neutralizing antibodies, which target VP2, confer protection against BTV infection and the subsequent development of bluetongue (BT) disease (5). BTV is transmitted by biting midges of the *Culicoides* species and infects domestic and wild ruminants resulting in outbreaks of BT disease, which cause significant economic and agricultural burdens (6). Traditionally, the prevalence of BT disease was restricted to tropical, subtropical, and temperate regions favored by its *Culicoides* vector; however, the advent of global warming has seen the expansion of vector distribution northwards, resulting in outbreaks of BT disease in northern Europe (7). The BTV8 outbreak in 2006–2008 was the single largest BTV outbreak ever recorded in Europe, with over 2 million animals infected, the majority of which were sheep. However, BTV8 was also found to be virulent in cattle, resulting in atypical symptomatic BT disease and mortality (8).

At least 28 BTV serotypes have been identified to date based upon unique VP2 neutralizing antibody profiles. The antigenic relatedness of BTV serotypes is by large reflected in their nucleotype groupings based upon nucleotide sequence similarity (3, 9). The neutralizing antibody responses, which target VP2 epitopes, are predominantly serotype specific, although evidence of sporadic, low-level, cross-neutralization between heterologous serotypes has been observed in vaccine trials and for both monoclonal antibodies (MAbs) and polyclonal antisera tested in tissue culture neutralization assays (9–11). Cross-reactivity, based upon antibody binding, is a more frequent occurrence, and in some instances, the magnitude of the VP2 antibody response against the heterologous serotype matches that of the homologous serotype (9–11). This implies that different serotypes share common VP2 epitopes; however, these epitopes are either non-neutralizing for both serotypes or non-neutralizing in the conformational context of the heterologous VP2 protein.

Antigenic studies of VP2 have been carried out for BTV1, BTV10, and BTV17 through the selection of neutralization-resistant viral variants or escape-mutant viruses (EMVs), followed by sequence analysis to identify amino acid mutations in the VP2 protein, which conferred the resistance to, or escape from, MAb neutralization (12–17). Additional studies, utilizing peptide mapping, have identified linear B-cell epitopes in the VP2 proteins of BTV1, BTV13, BTV16, and BTV25 (18–22). The majority of amino acid residues critical for neutralizing MAb recognition are clustered in two regions of the VP2, designated Region 1 (R1), which spans Ala^199^ to Gln^213^, and Region 2 (R2), which spans Pro^321^ to Ala^346^ for BTV1 (16).

The atomic structure of the VP2 identified four structural domains, which make up each monomer: the hub, hairpin, body, and tip (23). The hub domains of three monomers associate to form the triskelion-like trimers, which project from the outer capsid of BTV. The tip domain (designated as Pro^191^ to Ile^407^ for BTV1) (23) at the apex of the VP2 monomer is prominently positioned for both immune surveillance and the binding of antibodies, which would disrupt the attachment and entry into the host cells (2). Using this earlier atomic structure of VP2, the R1 and R2 could be positioned within the tip domain. However, the exact location of these regions and the residues within them critical for neutralizing MAb recognition could not be mapped precisely since this atomic structure lacked a modeled tip domain.

In this current study, we took advantage of a recently available atomic model of VP2 complete with a modeled tip domain (24), in order to study the structural basis of VP2 serotype specificity. VP2 homology models of BTV1 and the highly virulent BTV8 were generated and used to predict structural differences between these two serotypes. These data informed the design and generation of a panel of mutant VP2 proteins and BTV viruses. The antigenicity of these constructs was dissected using a panel of mouse MAbs and a rabbit polyclonal antiserum generated against recombinant VP2 representing BTV1. The data generated contribute to our understanding of the impact of VP2 structure on antigenic differences between BTV serotypes, which demonstrated differential virulence during disease outbreaks.

## RESULTS

### Homologous reactivity of BTV1 anti-VP2 antibodies demonstrates a range of functional specificities

**Fig 1:**
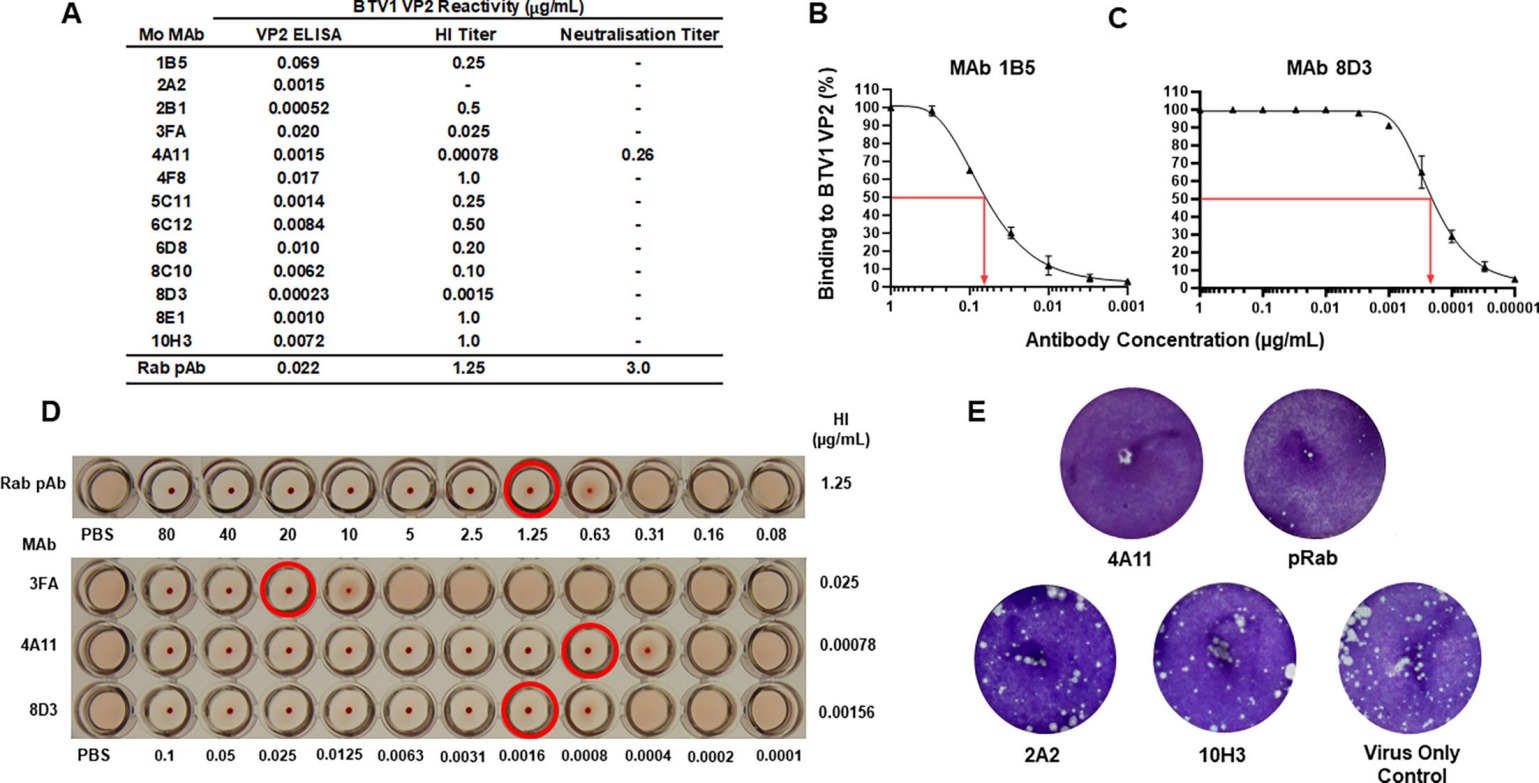
Homologous reactivity of a panel of BTV1 anti-VP2 antibodies. (**A**) BTV1 VP2 reactivity of antibody panel represented as 50% binding concentration in VP2 ELISA, 100% HI concentration, and 50% neutralization concentration. (-) indicates no antibody reactivity with a starting concentration of 1 µg/mL. Data represent the average concentrations derived from three to six replicates. BTV1 VP2 antibody binding profiles of a MAb 1B5 (**B**) and MAb 8D3 (**C**) with red arrows indicating 50% binding concentrations in µg/mL. Error bars represent standard deviation of replicates. (**D**) HI titers of the Rab pAb and three MAbs: 3FA, 4A11, and 8D3. Red circles indicate the antibody concentration resulting in 100% HI of multivalent BTV1 VP2 presented on nanoparticles. (**E**) Wells from neutralization assay of two non-neutralizing antibodies (2A2 and 10H3) and the two neutralizing antibodies (4A11 and Rab pAb), which inhibited plaque formation by BTV1 generated by reverse genetics. The virus-only control well demonstrates BTV1 plaque formation in the absence of antibody.

A panel of 13 mouse MAbs and a single rabbit polyclonal antiserum (Rab pAb) were raised against recombinant VP2 protein representing BTV1 (GenBank accession number: FJ969720). Mouse and rabbit IgG concentrations were determined in order to standardize antibody input, allowing direct comparisons across the range of assays utilized. The homologous reactivity of the antibody panel was assessed for binding, hemagglutination inhibition (HI), and neutralization activity against recombinant VP2 protein or virus generated via reverse genetics, representing BTV1. All antibodies bound BTV1 VP2 when tested in an indirect ELISA (Fig. 1A), with 50% antibody binding concentrations ranging from 0.069 µg/mL for MAb 1B5 (Fig. 1B) to 0.00023 µg/mL for MAb 8D3 (Fig. 1C).

The ability of the antibodies to inhibit the hemagglutination activity of VP2 was tested in a HI assay, which utilized recombinant VP2 protein bound to nanoparticles in order to increase the valency of VP2 presentation, as previously described (4). All antibodies were able to inhibit hemagglutination at a concentration of ≤1 µg/mL, except for MAb 2A2, which did not exhibit any inhibitory activity (Fig. 1A). The antibody concentrations required to achieve 100% inhibition of VP2 hemagglutination were higher than the 50% binding concentrations measured by ELISA, for example, the HI activity of MAbs 2B1, 4F8, 5C11, 8E1, and 10H3 required a >100-fold increase in antibody concentration. However, the three MAbs, 3FA, 4A11, and 8D3, which demonstrated the strongest HI activity, did so at antibody concentrations similar (≤7-fold difference) to those recorded for their VP2 binding (Fig. 1D). These data indicated that the epitopes targeted by MAbs 3FA, 4A11 and 8D3 directly overlap with the sialic acid binding site or impaired access to the site, through antibody-mediated conformational changes in VP2.

Only two antibodies, the Rab pAb and the MAb 4A11, exhibited neutralization activity, which targeted BTV1 (Fig. 1A), with 50% neutralization concentrations based upon plaque reduction of 3 µg/mL and 0.26 µg/mL, respectively. The remaining MAbs did not exhibit any neutralization activity, which targeted BTV1 when tested at a starting concentration of 1 µg/mL (Fig. 1E). These data demonstrated that the antibodies in the panel would be useful tools for the dissection of VP2 antigenicity since they exhibited different ranges of homologous reactivity, from simple recognition of BTV1 VP2, through to strong HI and neutralization activity.

### VP2 amino acid sequence underpins the differential antigenicity of heterologous BTV serotypes

**Fig 2:**
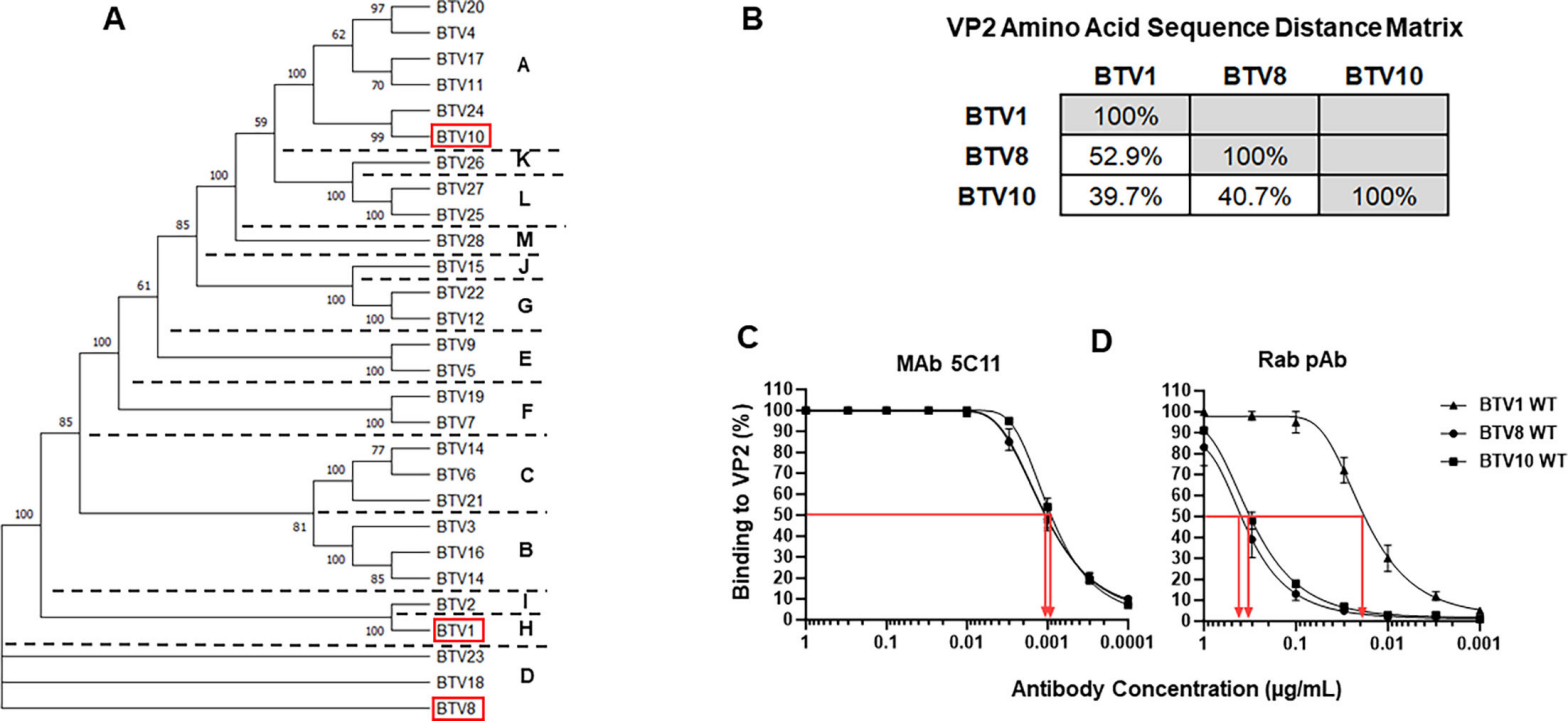
Heterologous VP2 sequence analysis and reactivity of BTV1 anti-VP2 antibodies. (**A**) A neighbor-joining phylogenetic tree constructed with VP2 amino acid sequences of BTV1 to BTV28, depicting groupings A–M. BTV1, BTV8, and BTV10 are highlighted. (**B**) Matrix presenting the VP2 amino acid sequence distances between BTV1, BTV8, and BTV10, as a percentage of identity. BTV1 (triangle), BTV8 (circle), and BTV10 (square) VP2 antibody binding profiles of MAb 5C11 (**C**) and Rab pAb (**D**) with red arrows indicating 50% binding concentrations in µg/mL. Error bars represent standard deviation of three to six replicates.

VP2 is the main serotype determinant of BTV, and antibody cross-reactivity is generally observed between serotypes, which cluster together based upon the phylogenetic analysis of VP2 sequence (Fig. 2A). In order to control for inter-serotype differences in VP2 amino acid sequence and their predicted impact upon protein structure, a BTV10 was included in the analysis alongside BTV1 and BTV8. All three serotypes fall into distinct groups (BTV1-H; BTV8-D; and BTV10-A) (Fig. 2A), which are underpinned by differences in VP2 amino acid sequence, with BTV8 and BTV10 having amino acid sequence homology to BTV1 of 52.9% and 39.7%, respectively (Fig. 2B). These data highlight that BTV10 is more distantly related to BTV1 than BTV8 based upon VP2 amino acid sequence analysis.

Recombinant VP2 proteins representing BTV8 and BTV10 (GenBank accession numbers: KJ872780 and AAA66972) were used as coating antigens in an indirect ELISA to assess whether the antibodies in the panel demonstrated cross-reactivity with either serotype. Two antibodies, the Rab pAb and the MAb 5C11, were able to recognized and bind both BTV8 and BTV10. The near-identical 50% antibody binding concentrations of MAb 5C11 against BTV1 (0.0012 µg/mL), BTV8 (0.0011 µg/mL), and BTV10 (0.00092 µg/mL) indicated that the VP2 epitope recognized by MAb 5C11 is highly conserved between all three serotypes (Fig. 2C). This was in contrast to the Rab pAb, where the 50% antibody binding concentrations against the heterologous VP2 proteins (BTV8, 0.468 µg/mL; BTV10, 0.351 µg/mL) were severalfold higher than against the homologous VP2 of BTV1 (0.020 µg/mL) (Fig. 2D). The majority (*n* = 12) of the antibody panel did not recognize either serotype, in line with serotype specificity of the antibody response to the VP2 of BTV.

### Heterologous VP2 homology models predict structural differences with potential antigenic impact

In order to explore how differences in protein structure between the serotypes correlated with the differences in VP2 antigenicity, pairwise comparisons were carried out between a BTV1 VP2 homology model and VP2 homology models representing BTV8 and BTV10. The superimposition of BTV8 and BTV10 homology models onto the BTV1 homology model generated Root Mean Squared (RMS) values of 0.32 Å and 0.42 Å, respectively. The difference in RMS values between the two models (BTV1:BTV8 and BTV1:BTV10) at each individual amino acid position of the VP2 protein was recorded and plotted, with amino acid positions predicted to have insertions or deletions given an arbitrary RMS value of 10. Differences in RMS were considered significant if they were equal to, or 10 times greater than, the RMS value generate by the pairwise comparisons of the homology models.

**Fig 3:**
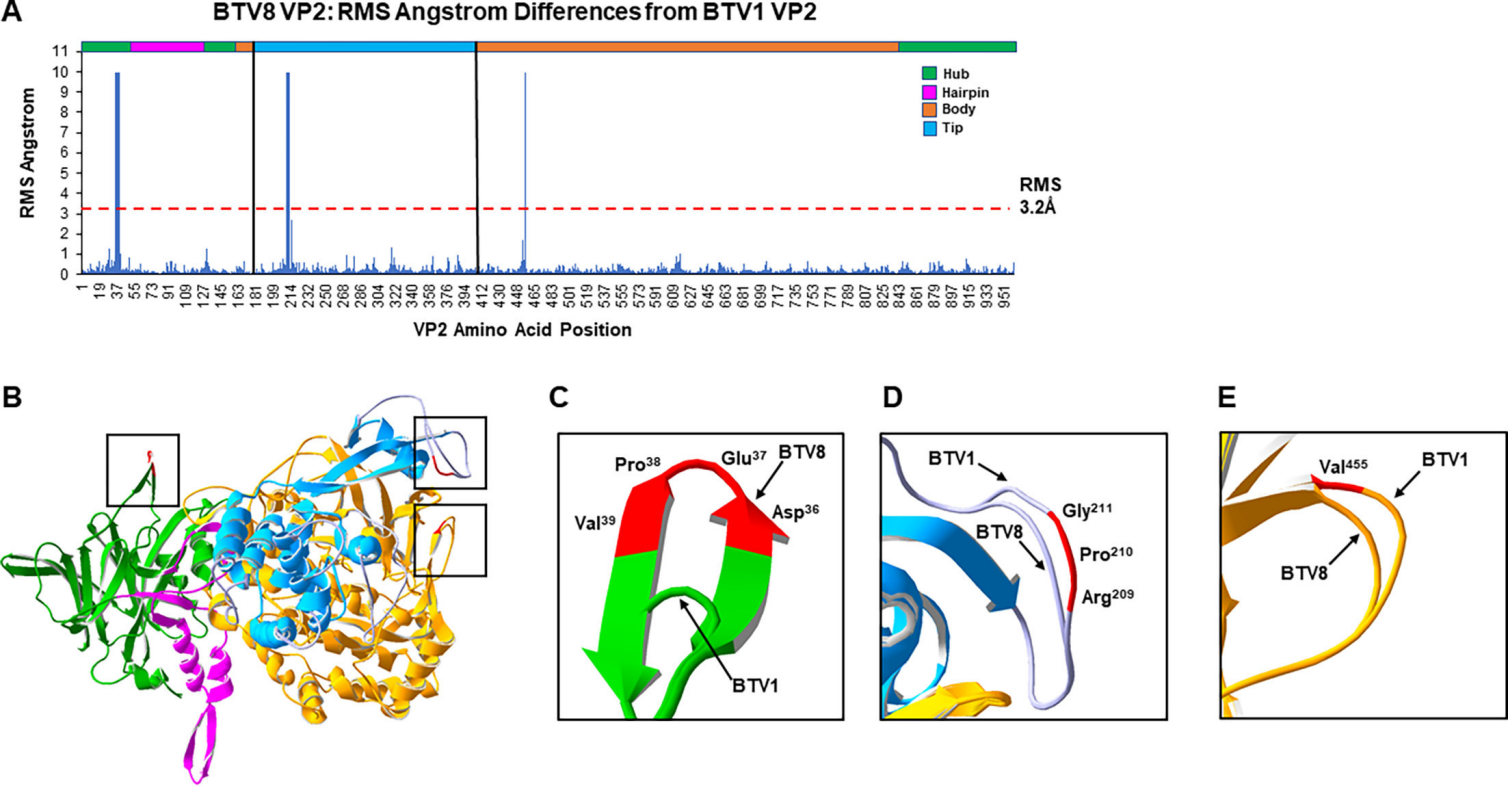
Predicted differences in VP2 structure between homology models representing BTV1 and BTV8. (**A**) Bar graph representing the RMS angstrom (Å) difference from BTV1 VP2 at each amino acid position of the BTV8 VP2 protein, as predicted from the superimposition of the VP2 homology model representing BTV8 onto BTV1. The color bar at the top is a linear representation of the domains in the VP2 protein based upon BTV1, with black vertical lines highlighting the tip domain. Red dash line indicates the total RMS value for the pairwise homology model, with individual amino acid RMS values above considered to be significant. (**B**) Side view of VP2 pairwise model generated by the superimposition of the BTV8 monomer onto BTV1, with the four structural domains of the monomers color coded. The hub domain is in green (Met^1^-Trp^49^, Gly^121^-Cys^162^, and Lys^839^-Val^961^), the hairpin is in pink (Asp^50^-Val^120^), the body is in orange (Leu^163^-Lys^190^ and Tyr^408^-Thr^838^), and the tip is in blue (Pro^191^-Ile^407^) (23). Predicted structural differences between BTV1 and BTV8 are highlighted. Expanded view of predicted structural differences in the hub domain (**C**), the tip domain (**D**), and the body domain (**E**). The positions of amino acids, which contribute to differences, are highlighted in red.

For the comparison between BTV8 and BTV1, three regions of BTV8 VP2 were predicted to be structurally different from the VP2 of BTV1 based upon an RMS value ≥ 3.2 Å (Fig. 3A). These regions were located in the hub, tip, and body domains of the VP2 monomer (Fig. 3B). The RMS peaks were the consequent of a four-amino acid insertion in the BTV8 hub domain compared with BTV1 (BTV8 residues: Asp^36^, Glu^37^, Pro^38^, and Val^39^) (Fig. 3C), a three-amino acid deletion in the BTV8 tip domain compared with BTV1 (BTV1 residues Arg^209^, Pro^210^, and Gly^211^) (Fig. 3D), and a one amino acid deletion in the BTV8 body domain compared with BTV1 (BTV1 residue Val^455^) (Fig. 3E).

**Fig 4:**
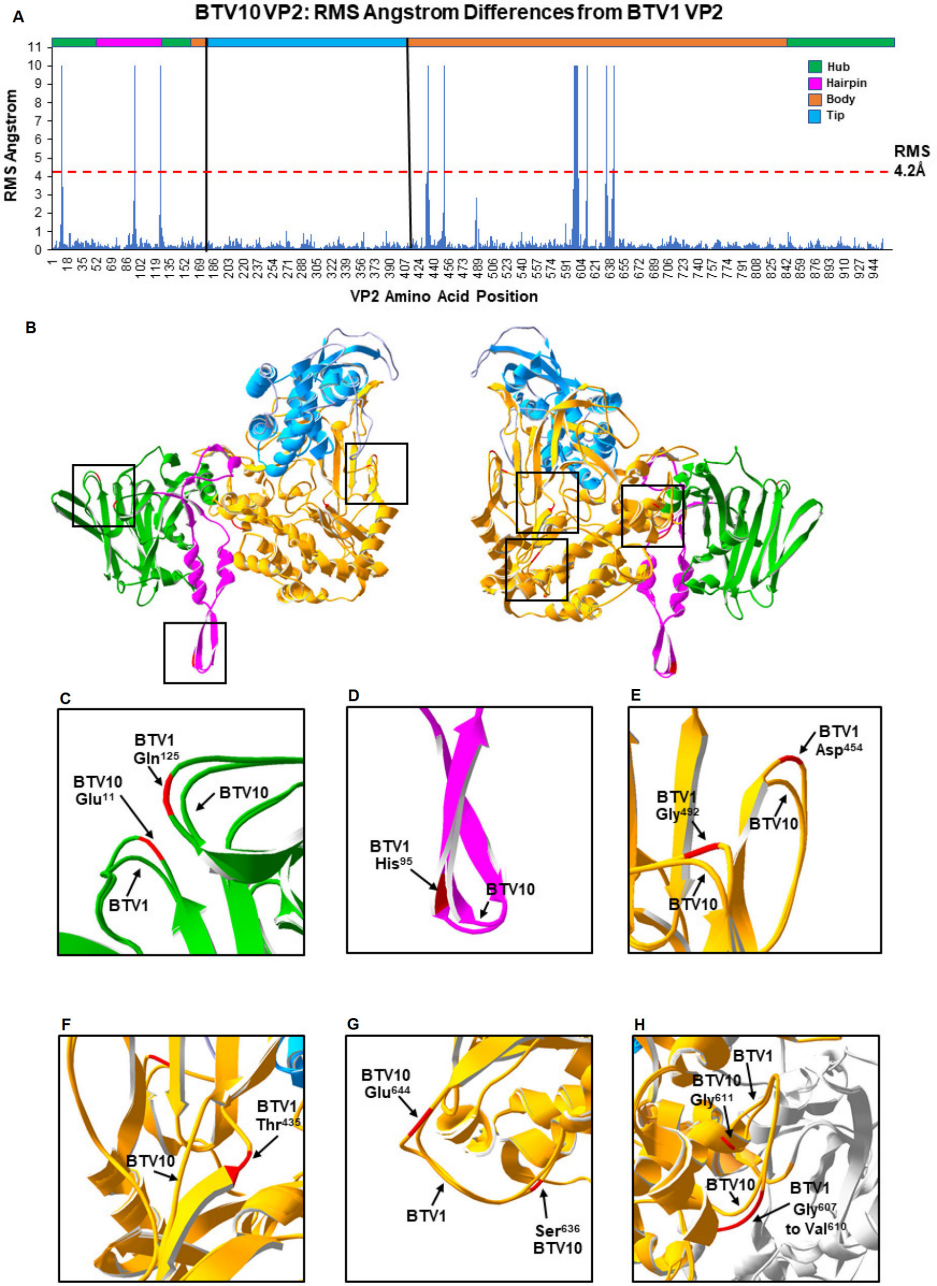
Predicted differences in VP2 structure between homology models representing BTV1 and BTV10. (**A**) Bar graph representing the RMS angstrom difference from BTV1 VP2 at each amino acid position of the BTV10 VP2 protein, as predicted from the superimposition of the VP2 homology model representing BTV10 onto BTV1. The color bar at the top is a linear representation of the domains in the VP2 protein based upon BTV1, with black vertical lines highlighting the tip domain. Red dash line indicates the total RMS value for the pairwise homology model, with individual amino acid RMS values above considered to be significant. (**B**) Side view and side view turned 180^o^ of VP2 pairwise model generated by the superimposition of the BTV10 monomer onto BTV1, with the four structural domains of the monomers color coded. Predicted structural differences between BTV1 and BTV10 are highlighted. Expanded view of predicted structural differences in the hub domain (**C**), the hairpin domain (**D**), and the body domain (E–H). The positions of amino acids, which contribute to differences, are highlighted in red.

In the comparison between BTV10 and BTV1, nine regions of BTV10 VP2 were predicted to be structurally different to the VP2 of BTV1 based upon an RMS value ≥ 4.2 Å (Fig. 4A). These regions were located in the hub, hairpin, and body domains of the VP2 monomer (Fig. 4B). In the hub domain, the RMS peaks were due to a single amino acid insertion (BTV10 residue Glu^11^) and deletion (BTV1 residue Gln^125^) in BTV10 compared with BTV1 (Fig. 4C). The peak in the hairpin domain was due to a deletion in BTV10 (BTV1 residues His^95^) relative to BTV1 (Fig. 4D). The numerous peaks in the body domain were due to multiple insertions (BTV10 residues Gly^611^, Ser^636^, and Glu^644^) and deletions in BTV10 (BTV1 residues Thr^435^, Asp^454^, Gly^492^, Arg^607^, Gly^608^, Ile^609^, and Val^610^) relative to BTV1 (Fig. 4E through H).

Taken together, these data indicate that the VP2 of BTV10 contains more predicted structural differences than the VP2 of BTV8, relative to the VP2 of BTV1. However, even though BTV10 had a higher number of differences, structural differences in the tip domain were only predicted between BTV8 and BTV1. The tip domain of VP2 is known to harbor neutralizing antibody epitopes; therefore, structural differences in this region may have a direct impact upon antigenicity.

### VP2 tolerates mutational manipulation of the tip domain

**Fig 5:**
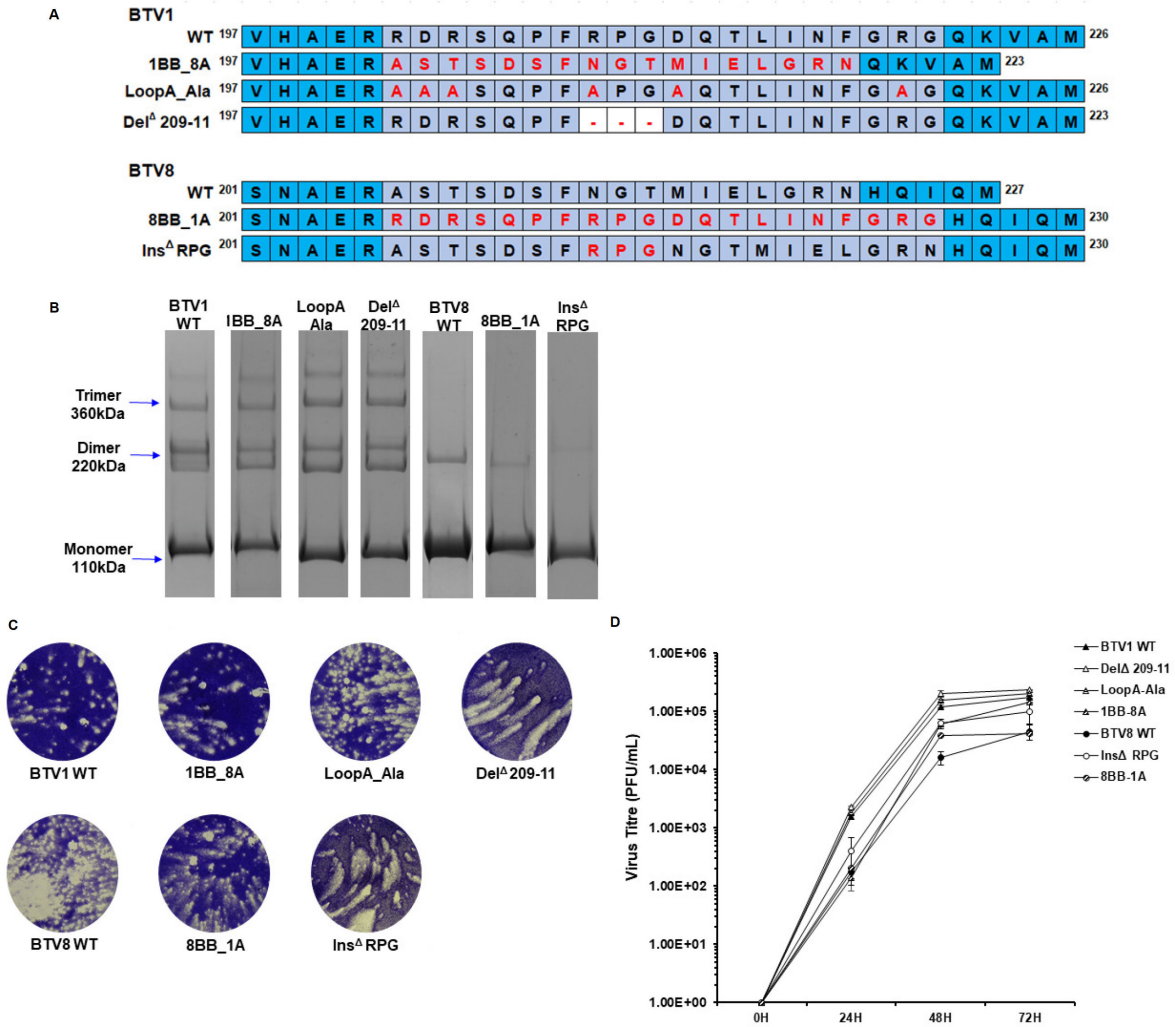
VP2 tip region loop A mutants. (**A**) BTV1 and BTV8 amino acid alignments of loop A (light-purple) with flanking regions (blue). Mutant loop A sequences are presented under the wild-type (WT) sequence of each serotype. Amino acid residues, which are changed relative to WT, are highlighted in red with deleted residues indicated by a red dash. (**B**) PAGE with Coomassie blue staining showing that the native VP2 proteins of the WT and mutant BTV1 and BTV8 constructs associate as dimers and trimers in the absence of heat or reducing reagent. (**C**) BTV reverse genetics containing WT or mutant S2 genes of BTV1 or BTV8. Plaque assay shows successful virus recovery of all viruses at 72 hours post-transfection. (**D**) Single-step growth curve of mutant viruses compared with their respective WT virus. BTV1 WT (filled triangle), Del^Δ^ 209-11 (empty triangle), LoopA_Ala (horizontal dashed triangle), and 1BB_8A (diagonal dashed triangle). BTV8 WT (filled circle), InsΔ RPG (empty circle), and 8BB_1A (diagonal dashed circle). Error bars represent standard deviation of two replicates.

The superimposition of the BTV8 VP2 homology model onto BTV1 highlighted a predicted structural difference between the two serotypes due to the deletion of three residues in BTV8 compared with BTV1 (Fig. 3D). This deletion was located in the first flexible loop region of the tip domain, designated loop A. In order to investigate whether this structural difference in loop A had an impact upon VP2 antigenicity, a panel of VP2 mutants was designed (Fig. 5A). These mutant constructs included the following: VP2 where loop A had been switched between BTV1 and BTV8 (1BB_8A & 8BB_1A), a BTV1 VP2 where all the charged residues in loop A had been switched to alanine residues (LoopA_Ala), a BTV1 VP2 with a three-amino acid deletion in loop A (Del^Δ^ 209-11), and a BTV8 VP2 with those same three amino acids inserted into loop A (Ins^Δ^ RPG). This mutant panel was designed with the aim of determining the overall contribution of loop A to VP2 antigenicity, in addition to interrogating the antigenic consequence of the structural difference in loop A between BTV1 and BTV8.

Recombinant BTV1 and BTV8 VP2 proteins harboring loop A mutations were successfully expressed using the baculovirus system. Staining with Coomassie blue confirmed the solubility and purity of each VP2 protein expression and PAGE analysis in the absence of β-mercaptoethanol, and heat treatment demonstrated dimer and trimer formation at levels comparable to the respective WT protein (Fig. 5B). BTV1 and dual reassorted BTV1/BTV8 viruses harboring mutations in the S2 gene were successfully rescued by reverse genetics (Fig. 5C). Single-step viral growth curves generated for all viruses demonstrated that the mutant viruses had similar growth phenotypes to the respective WT virus (Fig. 5D). Dual reassorted BTV1/BTV8 viruses were generated by replacing the S2 and S5 RNA segments of BTV1 with the corresponding RNA segments of BTV8, as previously described (25). The S2 segment, which encodes for VP2, either possessed the BTV8 WT sequence or the sequence representing the loop A mutants 8BB_1A and Ins^Δ^ RPG. The ability to successfully express VP2 proteins and rescue BTV viruses harboring mutations in the tip domain, even drastic manipulations such as inter-serotype loop swaps (1BB_8A and 8BB-1A), demonstrates the mutational tolerance of VP2.

### The loop A of BTV1 is an important antigenic target for anti-VP2 antibodies

**Fig 6:**
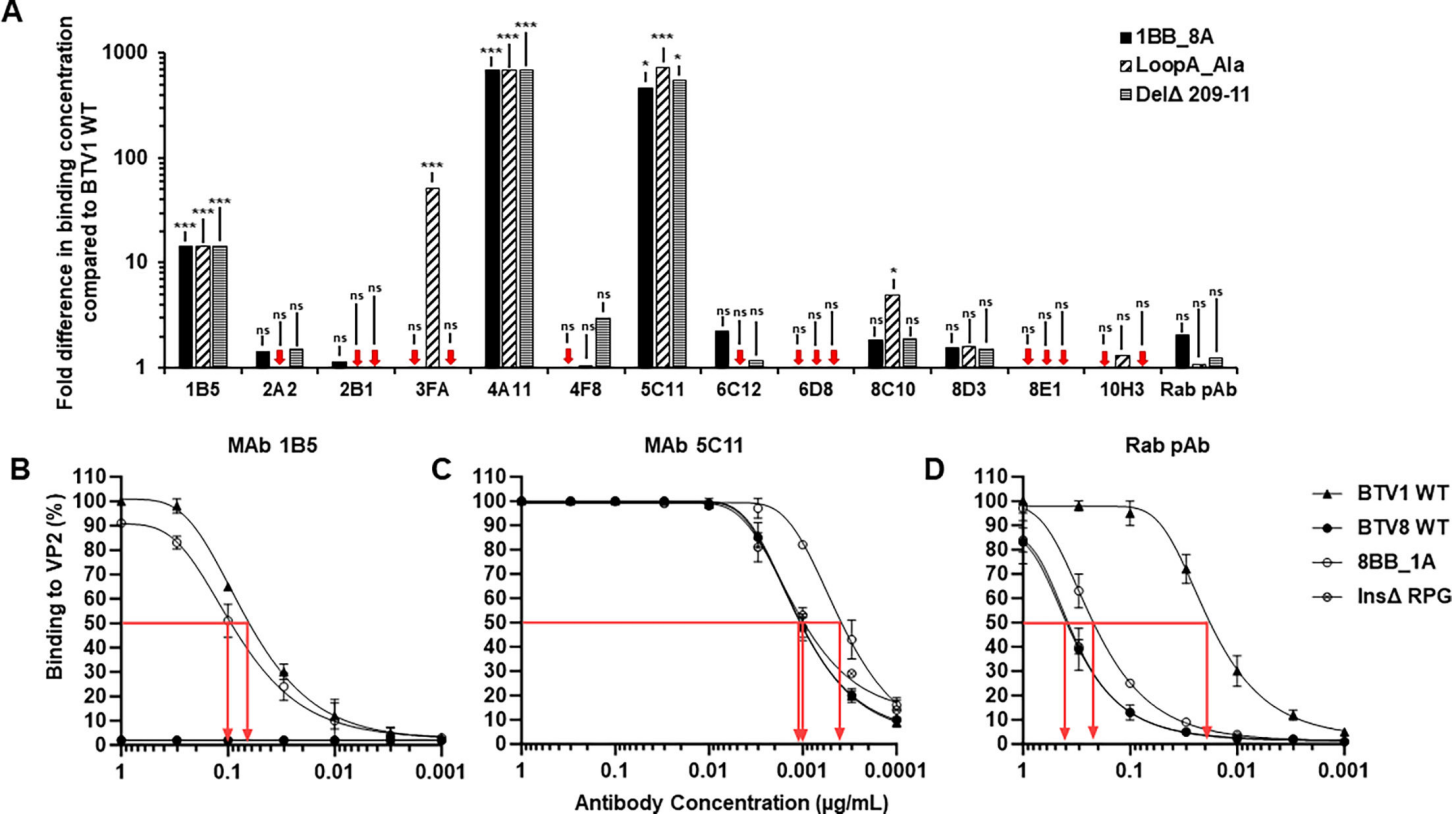
BTV1 anti-VP2 antibody binding to loop A mutants. (**A**) Bar graph representing the fold difference in the 50% binding concentrations compared with WT BTV1 VP2 for each antibody against each of the three BTV1 loop A mutants: 1BB-8A (filled bars), LoopA_Ala (diagonal dashed bars), and Del^Δ^ 209-11(horizontal dashed bars). Red arrows indicate a fold difference of <1. Statistical analysis: two-tailed unpaired t test (three to six replicates) ***P < 0.001, **P < 0.01, and *P < 0.05, ns *P* > 0.05. BTV1 WT (filled triangle), BTV8 (filled circle), 8BB_1A (empty circles), and Ins^Δ^ RPG (horizontal dashed circle) VP2 antibody binding profiles of MAb 1B5 (**B**), MAb 5C11 (**C**), and Rab pAb (**D**) with red arrows indicating 50% binding concentrations in µg/mL. Error bars represent standard deviation of three to six replicates.

The VP2 recombinant proteins harboring the loop A mutations 1BB-8A, LoopA_Ala, and Del^Δ^ 209-11 in a BTV1 backbone and 8BB_1A and Ins^Δ^ RPG in a BTV8 backbone were used as coating antigens in an indirect ELISA to assess whether the antibodies in the panel were able to recognize the mutants. The 50% binding concentration of each antibody against the BTV1 loop A mutants 1BB-8A, LoopA_Ala, and Del^Δ^209-11 was determined and used to calculate the fold difference in binding concentration relative to the BTV1 WT (Fig. 6A). Five MAbs, 1B5, 3FA, 4A11, 5C11, and 8C10, had a significant increase in 50% binding concentration against at least one of the loop A mutants compared with the BTV1 WT. The loop A mutants had no significant impact upon the binding of the remaining eight antibodies in the panel, indicating that these antibodies recognize epitopes outside of the loop A region of VP2. Only three antibodies recognized the mutants with a BTV8 backbone, the MAb 1B5 (Fig. 6B), the MAb 5C11 (Fig. 6C), and the Rab pAb (Fig. 6D). The latter two antibodies had previously demonstrated recognition of WT BTV8 VP2. None of the other antibodies in the panel recognized the mutants with the BTV8 backbone (data not shown).

MAbs 3FA and 8C10 had a significant reduction in binding to the LoopA_Ala mutant relative to the BTV1 WT, requiring a 51-fold and 5-fold increase in antibody concentration, respectively, to achieved 50% binding against the mutant (Fig. 6A). Both MAbs were able to bind the 1BB_8A and Del^Δ^209-11 mutants indicating that charged amino acids, six of which are located in the loop A of BTV1, were required for epitope recognition but that the positively charged Arg^209^ absence in the Del^Δ^209-11 mutant was not critical for binding.

Three MAbs, 1B5, 4A11, and 5C11, had reduced or a complete loss of binding to all three loop A mutants. MAb 1B5 had the highest 50% binding concentration against the BTV1 WT of all the antibodies in the panel, and the loss of binding to the mutants could be a consequence of the relatively low avidity and affinity of this MAb. However, this MAb was able to recognize the BTV8 loop A mutant 8BB_1A (Fig. 6B), which had the loop A of BTV1 in a BTV8 VP2 backbone, but 1B5 did not recognize the Ins^Δ^ RPG mutant. These data indicated that the epitope of 1B5 encompasses residues in the loop A of BTV1 and that recognition of Arg^209^, Pro^210^, and Gly^211^ requires the complete loop A of BTV1.

The neutralizing MAb 4A11 was unable to bind any of the loop A mutants, irrespective of whether they were in a BTV1 (Fig. 6A) or BTV8 backbone (data not shown). Loss of binding to the BTV1 loop mutant Del^Δ^209-11 demonstrated that residues Arg^209^, Pro^210^, and Gly^211^ were potentially critical for 4A11 recognition and/or that the 4A11 epitope is highly conformation dependent and disrupted by the deletion. These data indicate that the 4A11 epitope encompasses residues in the loop A of BTV1, including ones which are predicted to result in VP2 structural differences between BTV1 and BTV8.

MAb 5C11, which binds a VP2 epitope conserved between BTV1, BTV8, and BTV10, had significantly reduced binding to the BTV1 loop A mutants (Fig. 6A). However, 5C11 binding to the BTV8 loop A mutants was observed with 50% binding concentrations comparable to concentrations recorded against WT VP2 representing both BTV1 and BTV8 (Fig. 6C). This pattern of binding indicates that in the context of a BTV1 backbone, mutations in loop A knocked out the binding of 5C11 to its epitope. However, in the context of a BTV8 backbone, mutations in the loop A have no effect upon 5C11 epitope binding. Taken together, these data would suggest that the 5C11 epitope is conformationally dependent and is located outside of, but within close proximity to, loop A. In addition, amino acid changes in loop A and the conformational pressures, which they exert upon the 5C11 epitope, are tolerated better in the BTV8 VP2 backbone compared with the BTV1 backbone.

The neutralizing Rab pAb, which exhibited cross-reactivity against BTV8 and BTV10, demonstrated no significant difference in the 50% bind concentration against the BTV1 loop A mutants due to its polyclonal nature (Fig. 6A). The binding profile for the BTV8 Ins^Δ^ RPG mutant was nearly identical to the binding profile against BTV8 WT (Fig. 6D); however, increased binding was observed against the 8BB_1A mutant. This indicated that a proportion of the polyclonal antibody response against BTV1 targets residues in the loop A of VP2.

### Anti-VP2 neutralizing antibodies target the loop A of BTV1

The two antibodies, which exhibited neutralization activity against BTV1, the Rab pAb and MAb 4A11, were tested for their ability to neutralize the loop A mutants. Neither antibody exhibited cross-neutralizing activity against BTV8 WT or loop A mutants in a BTV8 backbone (data not shown). The Rab pAb had previously demonstrated antibody specificities, which cross-reacted with recombinant VP2 representing BTV8 (Fig. 2D); however, these specificities appear not to target neutralizing epitopes.

**Fig 7:**
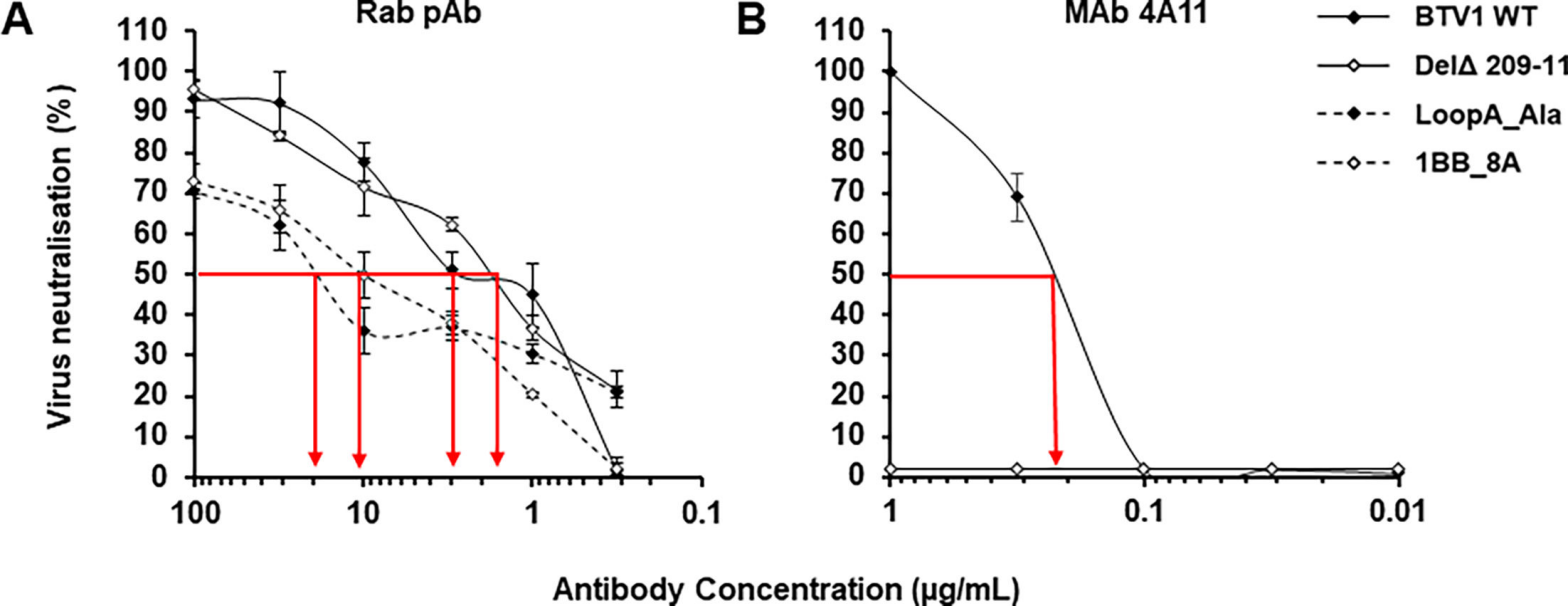
Anti-VP2 neutralizing antibody activity against loop A mutants BTV1 WT (filled diamonds), Del^Δ^ 209-11 (empty diamonds), LoopA_Ala (filled diamonds with dashed line), and 1BB_8A (empty diamonds with dashed line) VP2 neutralizing antibody binding profiles of Rab pAb (**A**) and MAb 4A11 (**B**) with red arrows indicating 50% neutralization concentrations in µg/mL. Error bars represent standard deviation of three to four replicates.

The Rab pAb was able to neutralize all the BTV1 loop A mutants but to different degrees (Fig. 7A), exhibiting a 50% neutralization concentration against the Del^Δ^ 209-11 mutant of 1.5 µg/mL compared with 3 µg/mL against the BTV1 WT. Higher antibody concentrations of 22.4 µg/mL and 11.9 µg/mL were required for the 50% neutralization of the LoopA_Ala and 1BB_8A mutants, respectively. These data demonstrate that charged residues are important for neutralizing antibody binding and that a proportion of the polyclonal neutralizing antibody response against BTV1 VP2 targets epitopes within loop A. The ability of the polyclonal antibodies to neutralize the 1BB_8A mutant, albeit at a higher concentration, demonstrates that there are neutralizing epitopes outside of loop A but that a lower proportion of neutralizing antibody specificities targets these epitopes. The neutralization activity of MAb 4A11 was completely lost when tested against the loop A mutants (Fig. 7B). These data demonstrated that the loop A of BTV1 contains a serotype-specific neutralizing epitope and that critical residues within the epitope (Arg^209^, Pro^210^, and Gly^211^) are positioned in the loop A region, predicted to be structurally different between BTV1 and BTV8.

**Fig 8:**
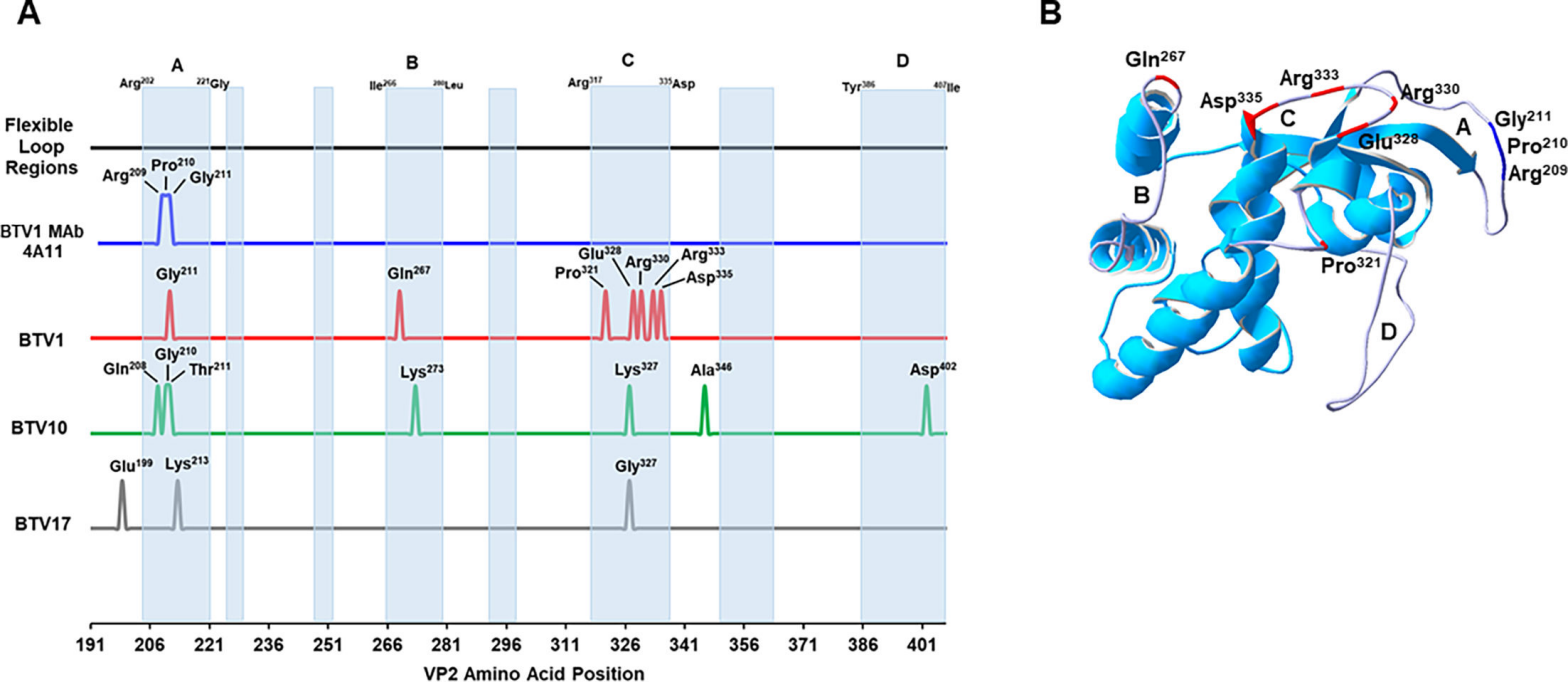
Location of VP2 amino acid residues critical for neutralizing antibody recognition. (**A**) VP2 amino acid position of residues critical for the neutralizing antibody recognition of BTV1 (red), BTV10 (green), and BTV17 (gray), extracted from previously published data (12–17), was compared with the position of the loop A residues Arg^209^, Pro^210^, and Gly^211^ critical for BTV1 neutralization by MAb 4a11 (blue). Amino acid positions are indicated by peaks. The VP2 regions with predicted flexibility and the potential to form loops (light-blue shading) are indicated, with the four largest regions designated A (Arg^202^-Gly^221^), B (Ile^266^-Leu^280^), C (Arg^217^-Asp^335^), and D (Tyr^386^-Ile^407^). (**B**) Expanded view of the BTV1 VP2 tip domain (Pro^191^-Ile^407^), with the flexible loop regions A to D, colored in light-purple. The position of amino acids critical for BTV1 neutralizing antibody recognition is highlighted on flexible loop regions A, B, and C.

The availability of the tip domain structure allowed the residues critical for MAb 4A11 BTV1 neutralization to be mapped to the loop A region. To determine whether the flexible loop regions of the tip domain are hot spots for residues that are critical for neutralizing antibody recognition, the positions of any VP2 residues in the tip domain, which when mutated resulted in the loss of antibody-mediated virus neutralization, were extracted from previously published studies (12–17) and mapped on to the tip domain (Fig. 8A). This analysis demonstrated that the loop A of VP2 is a hotspot for residues, which contribute to neutralizing antibody epitopes across the three serotypes, BTV1, BTV10, and BTV17, for which data were available. The tip domain of the VP2 has flexible loop regions interspersed between the secondary structural features (Fig. 8B), and the four largest regions, designated loops A to D, contain most residues critical for neutralizing antibodies (Fig. 8A). In BTV1, critical residues cluster together at the apex of the tip domain in loops A and C, with a single residue mapped to loop B (Fig. 8B).

## DISCUSSION

This study investigated the inter-serotype VP2 structural differences, which underpin the differential antigenicity of BTV1 and the highly virulent BTV8. The atomic model of VP2 with a complete tip domain (24) was utilized as the structural template for the generation of VP2 homology models representing BTV1 and BTV8. Data from this analysis were used to predict structural differences between serotypes, which informed the design and generation of mutant VP2 constructs. The antigenicity of these mutant constructs was characterized alongside the wild type (WT) using a panel of anti-VP2 antibodies raised against BTV1.

All the mouse MAbs and the rabbit polyclonal antiserum were able to bind to BTV1 VP2; however, differential HI and neutralization activity was exhibited. The hemagglutination activity of VP2 in recombinant form was initially demonstrated using sheep erythrocytes with activity inhibited by anti-VP2 antibodies (2), suggesting that sialic acid motifs may play a role in the initiation of BTV attachment to the host cell. Subsequent work confirmed that VP2 binds to α2,3- and α2,6-linked sialic acids and mapped the potential sialic acid binding site in the VP2 to a linear sequence of amino acids, which spans the hub domain and the start of the tip domain (Val^185^ to Asp^194^ for BTV1) (4). In this current study, all the antibodies except one demonstrated HI activity at concentrations ≤ 1 µg/mL; however, only two antibodies demonstrated neutralization activity against BTV1 at the same starting concentration. These findings indicate, that for BTV, the HI titer cannot be used as a surrogate for protection against infection, as is the case for influenza virus where a HI titer of 1:40 is considered to provide a 50% reduction in the risk of acquiring the infection in adults (26). The ability of some antibodies to inhibit hemagglutination in the absence of neutralization activity implies that sialic acid binding is just one of the steps involved in BTV attachment and host cell entry.

VP2 is the main serotype determinant of BTV, and distinct serotypes are defined as having an amino acid sequence difference in the VP2 of >23% (3). BTV serotypes cluster into nucleotype groups based upon their VP2 nucleotide phylogenetic relationship with robust antibody cross-reactivity observed at a higher frequency within nucleotype groupings (9). VP2 homology models were generated for BTV1 and BTV8, alongside BTV10, by mapping the VP2 amino acid sequence of each serotype onto the atomic model (24). Pairwise comparison of the BTV1 homology model with the models generated from BTV8 and BTV10 predicted regions of structural divergence between serotypes. The number of predicted structural difference across the whole of the VP2 was highest in the pairwise comparison between BTV1 and BTV10, in line with the lower amino acid sequence identity (39.7%) between these two serotypes, but all the changes were outside of the tip domain, which is known to harbor neutralizing antibody epitopes (16). BTV1 and BTV8 have a higher amino acid sequence identity (52.9%), and the pairwise comparison of homology models was predicted to have fewer structural difference across the VP2; however, one of these differences was located in the tip region and, as such, had the potential to impact VP2 antigenicity. These data demonstrate the utility of homology models for providing additional information not apparent from the phylogenetic analysis of amino acid sequences.

VP2 exists as triskelion-like trimers on the outer capsid of the BTV particle, with the trimerization occurring through the interaction of the hub domains from three monomers (23). The external tip domain of the VP2 projects upwards away from the capsid surface and is well positioned to initiate virus contact with the host cell plasma membrane. The exposed nature of this domain is consistent with its role as the predominant target of the host antibody response (2). The recent VP2 cryo-EM structure included the previously unmodeled tip domain (24), allowing secondary structures and regions of flexibility within the tip domain to be mapped. Four surface-exposed flexible regions, which lacked secondary structure, were designated as loops A to D. Loop A, which is positioned at the apex of the tip domain, is the location of the predicted structural difference between BTV1 and BTV8. The region spanning the loop A of BTV1 contains 20 amino acid residues (Arg^202^ to Gly^221^) compared with 17 residues in BTV8 (Ala^206^ to Asn^222^), with the additional three amino acid residues extending the loop A of BTV1 relative to the loop A of BTV8. BTV1 and BTV8 pairwise amino acid sequence alignment and homology model comparisons identify the three additional BTV1 residues as Arg^209^, Pro^210^, and Gly^211^.

Preliminary analysis of additional BTV serotypes demonstrated that serotypes within the same group appear to possess tip domains with similar predicted structures, particularly in the flexible loop regions. For example, in group D, which contains BTV8, BTV18 and BTV23 also have the same three amino acids deleted in loop A, which results in a shortening of the loop relative to BTV1. This intra-group maintenance of loop structures can also be seen in group E, which contains BTV5 and BTV9. Both serotypes have a three-amino acid deletion, Thr^393^, Ala^394^, and Ala^395^, in loop D, which again results in a predicted shortening of the loop region relative to the loop D of BTV1 (data not shown). This sheds light on the potential structural foundation, which supports cross-neutralizing antibody recognition between serotypes within nucleotype groups but impedes the generation of a robust inter-nucleotype group cross-neutralizing antibody response (9–11).

Mutant VP2 proteins with loop A swaps, alanine replacement of charged residues and deletions (Arg^209^, Pro^210^, and Gly^211^), were used to determine the overall importance of loop A for BTV1 antigenicity. Nine of the antibodies in the panel recognized non-loop A epitopes in the VP2, in agreement with previous studies, which have mapped residues critical for antibody recognition to multiple sites across the tip domain (16). Of the remaining five antibodies, the BTV1 loop A mutants had the biggest impact on VP2 recognition by MAbs 4A11 and 5C11. The ability of MAb 4A11 to bind VP2 and neutralize BTV1 was completely lost against all three of the BTV1 loop A mutants. The minimum mutation, which resulted in the loss of 4A11 recognition, was the deletion of residues 209 to 211, demonstrating that the same residues, which are critical for the neutralizing epitope of 4A11, are also predicted to result in the loop A structural differences between BTV1 and BTV8. Taken together, these data provided insights into the structural differences in VP2, which underpin the serotype-specific neutralizing antibody response to BTV.

The MAb 4A11 exhibited the strongest HI activity and binds to amino acids in loop A directly downstream of the putative sialic acid binding site (4). Whether BTV1 neutralization is a direct result of 4A11 blocking access to this putative binding site or whether binding of 4A11 to loop A induces a conformational change in the VP2, which occludes the binding site, is not known. Conformational changes induced by neutralizing antibody binding have been reported for the receptor binding proteins of both enveloped and non-enveloped viruses (27, 28). A higher concentration of 4A11 was need to neutralize BTV1 (0.26 µg/mL) compared with the amount, which was required to inhibit the hemagglutination activity of the VP2 protein (0.00078 µg/mL). This may be due to the context in which VP2 is presented, either on the surface of a virus particle or as an individual recombinant protein bound to a nanoparticle. Alternatively, this difference in antibody concentration may be representative of the MAb valency required for neutralization versus HI, which would again indicate the involvement of multiple VP2 interactions during the process of host cell attachment and entry.

The MAb 5C11 recognizes a VP2 epitope, which is conserved across serotypes BTV1, BTV8, and BTV10. Binding of 5C11 to this conserved epitope does not neutralize BTV1, but it does inhibit the hemagglutination activity of BTV1 VP2. Epitopes which are conserved across serotypes generally elicit cross-reactive but not cross-neutralizing antibodies and as a consequence escape the evolution pressure of the immune response (29). Alternatively, epitopes are conserved due to their location in protein domains, which are critical to viral structure and/or function (30). The loop A mutants in a BTV1 VP2 backbone caused a dramatic reduction in 5C11 binding; however, no effect on binding was observed when the mutants were presented in a BTV8 VP2 backbone. This binding pattern indicates that 5C11 recognizes a conformationally dependent epitope, which is within close proximity of loop A. It also provides insight into mutational tolerance of VP2 from different serotypes since the 5C11 epitope was disrupted by the loop A mutants in a BTV1 backbone but was maintained in the BTV8 backbone. The ability of the influenza virus hemagglutinin protein to tolerant mutation contributes to the virus’s antigenic evolution (31). To a lesser extent, the BTV VP2 protein must exhibit mutational tolerance in order to support the evolution of serotypes and intra-serotype regional variants (32).

In this study, due to the absence of experimentally resolved crystal or atomic VP2 structures representing BTV8 and BTV10, homology models were constructed for the comparative structural analysis. Homology modeling is a standard approach, which has been used to predict the location of antigenic domains and sites of protein-protein interactions for viruses including H1N1 pandemic flu (33), HBV (34), and HPV (35). Additionally, only a panel of antibodies raised against VP2 representing BTV1 was available, which limited the scope of this study to the dissection of antigenicity in the context of BTV1. Access to BTV8 anti-VP2 antibodies would have allowed the study of the reciprocal antigenicity of BTV8, providing further insights into the factors that underpin serotype-specific antibody responses.

The differential HI and neutralization activity profiles of the individual MAbs tested in this study prompt questions about VP2 host cell attachment and entry and whether this is a multi-step, conformationally dynamic process. This has been seen with many other viral receptor binding proteins including the spike protein of SARS-CoV-2 (36), both capsid proteins of HPV (37) and gp120 of HIV-1 (38). It would be interesting to undertake cryo-EM studies of BTV or recombinant VP2 complexed with antibody in order to attempt to determine whether altered VP2 states do exist and to gain a better understanding of epitope-paratope recognition.

This study has demonstrated, for the first time, how structural differences in the tip domain of VP2 form the basis of the serotype-specific neutralizing antibody response to BTV. These structural differences between BTV1 and BTV8 were mapped to loop A, which sits at the apex of the VP2 tip domain and appears to be a hotspot for residues, which contribute to neutralizing antibody epitopes. These data increase our understanding of VP2 antigenicity and may be useful for guiding the *in silico* design of an epitope-based pan-reactive BTV vaccine.

## MATERIALS AND METHODS

### Generation of anti-VP2 antibodies

The rabbit and mouse immunizations were outsourced to a commercial company (Covalab S.A.S., Bron, France). The polyclonal antibody was generated by the immunization of two female New Zealand White rabbits using an 88-day protocol with recombinant VP2 representing BTV1 (GenBank accession number: FJ969720). Briefly, three intradermal injections of 500 µg of antigen mixed with incomplete Freund adjuvant were performed on days 0, 21, and 42. On day 63, a final boost by subcutaneous injection was performed with 500 µg of antigen. Final bleeds taken on day 88 were subjected to purification on a protein A column, and the final concentration of the purified polyclonal antibody was determined by reading the optical density at 280 nm using a UV spectrophotometer. Mouse MAbs were generated by the immunization of four female BALB/c mice using a 90-day protocol with recombinant VP2 representing BTV1. Briefly, five intradermal injections of 100 µg of antigen mixed with incomplete Freund adjuvant were performed on days 0, 14, 28, 49, and 63. On day 90, the spleen from one mouse was selected to undergo fusion with a myeloma cell line with the resulting hybridoma cells cultured in HAT-selective medium. Supernatants from 13 positive hybridoma clones were subsequently harvested. The mouse IgG concentration of the hybridoma supernatants was determined using a Mouse IgG ELISA Kit (Abcam) following the manufacturer’s instructions.

### Recombinant protein expression and purification

Recombinant baculovirus expressing WT or mutant VP2 representing BTV1, BTV8, and BTV10 (GenBank accession numbers: KJ872780 and AAA66972) was generated by co-transfecting the transfer vector pAcYM1-Strep-VP2 and Bacmid DNA into *Spodoptera frugiperda* (*Sf*9) cells. Sf9 cells were then infected with recombinant baculovirus and incubated at 28°C for 48 hours before cells were pelleted and lysed in buffer (100 mM Tris-HCl pH 8.0, 150 mM NaCl, 1 mM EDTA, 1% NP-40, and 1% Halt Protease Inhibitor Cocktail). Strep-VP2 protein was purified from the resulting lysate using Strep-Tactin Superflow high-capacity resin (IBA Lifesciences). BTV1 VP2 tagged with the Fc sequence from human IgG and a streptavidin tag II was expressed and purified in the same manner except the transfer vector pAcYM1-Strep-Fc-VP2 was used to generated the recombinant baculovirus. HEK293 cells were transfected with pA-LS, lumazine synthase (LS) nanoparticles with N-terminus protein A (pA) domains, and streptavidin tag II (4), using polyethylenimine. Supernatants were collected following incubation for 5 days, and Strep-pA-LS was purified using Strep-Tactin Superflow high-capacity resin.

### Recovery of BTV1 and dual reassorted BTV1/BTV8 viruses

Dual reassorted BTV1/BTV8 viruses were generated by replacing the S2 and S5 segments of BTV1 with the corresponding segments of BTV8 (GenBank accession numbers: KJ872780 and KJ872781). RNA transcripts were prepared from 10 individual pUC19 plasmids with T7 promoters containing BTV genome segments (S1–S10), using the mMESSAGE mMACHINE T7 Transcription Kit (Thermo Fisher Scientific) following the manufacturer’s instructions. BTV1 or dual reassorted viruses were recovered from confluent monolayers of BSR cells (BHK-21 [C-13], ATCC) after transfection with a full set of BTV T7 transcripts as previously described (39). Individual plaques were picked and amplified, and virus stocks were kept at 4°C.

### Mutagenesis of BTV segment 2

The loop A mutations were introduced into segment 2 of BTV1 or BTV8, in either the pUC19 plasmid with the T7 promoter or the pAcYM1 transfer plasmid, by site-directed mutagenesis. Mutations were confirmed by Sanger sequencing. The primer sequences used for mutagenesis were as follows: BTV1 - Del^Δ^ 209-11_FOR 5′-CGT AGT CAA CCG TTT GAT CAG ACA TTA ATT-3′ and Del^Δ^ 209-11_REV 5′-AAT TAA TGT CTG ATC AAA CGG TTG ACT TGC-3′, LoopA_Ala_FOR 5′-GCA GCG GCT GCC GCA GCG GCT GCC GCA GCG CAG AAG GTG GCA ATG ACC C-3′ and LoopA_Ala_REV 5′-GGC AGC CGC TGC GGC AGC CGC TGC GGC AGC CCT TTC AGC GTG GAC CAC-3′, 1BB_8A_FOR 5′-AAC TAT GAT CGA ATT AGG TCG TAA TCA GAA GGT GGC AAT GAA CC-3′ and 1BB_8A_REV 5′-CCA TTA AAG GAA TCA CTC GTT GAT GCC CTT TCA GCG TGG ACC AC-3′; BTV8 - Ins^Δ^ RPG_FOR 5′-ACG AGT GAT TCC TTT AGG CCA GGG AAT GGA ACT ATG ATC-3′ and Ins^Δ^ RPG_REV 5′-GAT CAT AGT TCC ATT CCC TGG CCT AAA GGA ATC ACT CGT-3′, 8BB_1A_FOR 5′-GAT CAG ACA TTA ATT AAT TTT GGG AGA GGT CAT CAA ATT CAG ATG GGT G-3′ and 8BB_1A_REV 5′-CCC TGG CCT AAA CGG TTG ACT ACG ATC TCT ACG TTC CGC ATT TGA TAT TAT C-3′.

### VP2 indirect ELISA

Nunc Maxisorp flat-bottom 96-well plates were coated overnight at 4°C with VP2 diluted in tris-buffered saline (TBS) at 50 ng per well. Wells were washed with 300 µL of wash buffer Tris-buffered saline with 0.05% Tween 20 (TBST-TBS and 0.05% Tween 20) and blocked at room temperature for 2 hours with 300 µL of blocking buffer (TBST, 5% non-fat milk) followed by three washes. Antibody samples were subjected to six to eight 1/2 Log_10_ serial dilutions carried out in sample buffer (TBST, 0.5% non-fat milk) before 50 µL was added to wells and incubated for 1 hour at 37°C. The wells were washed three times before a further incubation at 37°C for 1 hour with 50 µL of goat anti-rabbit or anti-mouse horseradish peroxidase-conjugated secondary antibody (Abcam) diluted in sample buffer. A final three washes preceded detection using 1-Step Ultra TMB (Thermo Scientific) according to the manufacturer’s instructions with absorbance read at 450 nm using the SpectraMax iD5 plate reader (Molecular Devices). The antibody concentration derived using 50% maximal binding optical density was estimated by interpolation.

### Hemagglutination inhibition assay

Purified Fc-VP2, 2.5 µg, was incubated with 1 µg of pA-LS for 30 min at room temperature to allow binding of Fc-VP2 to the pA-LS nanoparticles. Antibody samples were subjected to 11 twofold serial dilutions [carried out in an assay diluent of 0.1% bovine serum albumin in phosphate-buffered saline (0.1% BSA:PBS)]. In a 96-well V-bottom plate, the twofold serial dilutions of antibody were incubated with pA-LS-VP2 for 1 hour at room temperature to allow antibody binding to VP2 before the addition of a 0.5% suspension of wash sheep erythrocytes (Thermo Scientific, Oxoid) and incubation overnight at room temperature. Inhibition of hemagglutination was visualized as the formation of a distinct red pellet, and HI titers were calculated as the reciprocal of the lowest antibody concentration, which produced 100% inhibition of hemagglutination. Assay diluent was used as the negative control.

### Neutralization assay

The assay was performed as originally described (40) with some modifications. Antibody samples were diluted in unsupplemented Dulbecco’s modified Eagle’s medium before incubation with BTV standardized to an input of 100 PFU per reaction at room temperature for 1 hour. The antibody and BTV mixture was subsequently transferred to BSR cells and incubated for a further hour at room temperature before being removed. The cells were overlaid with 0.6% Avicel (IMCD) and incubated at 37°C for 72 hours. The BSR monolayers were fixed with 4% paraformaldehyde before staining with 1.2% crystal violet, and the plaques were counted. The results were expressed as the reciprocal of the antibody concentration that gave a 50% reduction in plaque number.

### Phylogenetic analysis of VP2

Full-length VP2 amino acid sequences representing serotypes BTV1 to BTV28 were downloaded from the NCBI database (BTV1: FJ969720; BTV2: AJ585123; BTV3: AJ585124; BTV4: AJ585125; BTV5: AJ585126; BTV6: AJ585127; BTV7: AJ585128; BTV8: KJ872780; BTV9: AJ585130; BTV10: AAA66972; BTV11: AJ585132; BTV12: AJ585133; BTV13: AJ585134; BTV14: AJ585135; BTV15: AJ585136; BTV16: AJ585137; BTV17: AJ585138; BTV18: AJ585139; BTV19: AJ585140; BTV20: AJ585141; BTV21: AJ585142; BTV22: AJ585143; BTV23: AJ585144; BTV24: AJ585145; BTV25: EU839840; BTV26: HM590642; BTV27: KM200718; and BTV28: MH559807). Using MEGAX version 10.1 (41), the VP2 amino acid sequences were aligned and analyzed using a Neighbor-Joining tree algorithm with the resulting phylogenetic tree supported by bootstrap values (*n* = 500 iterations).

### VP2 modeling

VP2 homology models were created from the VP2 amino acid sequences of BTV1, BTV8, and BTV10 using SWISS MODEL (http://swissmodel.expasy.org/) (42). The atomic structure of the BTV1 VP2 (Protein Data Bank code: 8W10) (24) was used as the template to which the target amino acid sequences were modeled. DeepView Swiss-Pdb viewer v4.1 (43) was used to perform pairwise VP2 homology model comparisons by superimposition, and predicted structural differences between models were measured in angstrom (Å).

### Statistical methods

GraphPad Prism version 10.0.3 (GraphPad Software, www.graphpad.com) was used to perform two-tailed unpaired t tests for the comparison of antibody titers between different antigen targets.

## Data Availability

The data that support the findings of this study are available upon request from the corresponding author (P.R.).
